# Supplementary material for: c-di-GMP inhibits rRNA methylation and impairs ribosome assembly in the presence of kanamycin
Source: EMBO Rep. 2025 Jan 27;26(5):1367–84. doi: 10.1038/s44319-025-00377-w (PMC11894153; doi:10.1038/s44319-025-00377-w)
Supplement: Supplementary file 1 — Appendix [file 44319_2025_377_MOESM1_ESM.pdf]

Appendix for

c-di-GMP modulates ribosome assembly by inhibiting rRNA methylation

Siqi Yu and Zheyao Hu, *et al*.

*Corresponding author. Email: xuzw@fjmu.edu.cn

Table of Contents

Appendix Figure S1……………………………………………………3
Appendix Figure S2……………………………………………………4
Appendix Figure S3……………………………………………………5
Appendix Figure S4……………………………………………………6
Appendix Figure S5……………………………………………………7
Appendix Figure S6……………………………………………………8
Appendix Figure S7……………………………………………………9
Appendix Figure S8……………………………………………………10
Appendix Figure S9……………………………………………………11
Appendix Figure S10…………………………………………………12
Appendix Figure S11…………………………………………………13
Appendix Figure S12…………………………………………………14
Appendix Figure S13…………………………………………………15
Appendix Figure S14…………………………………………………16
Appendix Figure S15…………………………………………………17
Appendix Figure S16…………………………………………………18

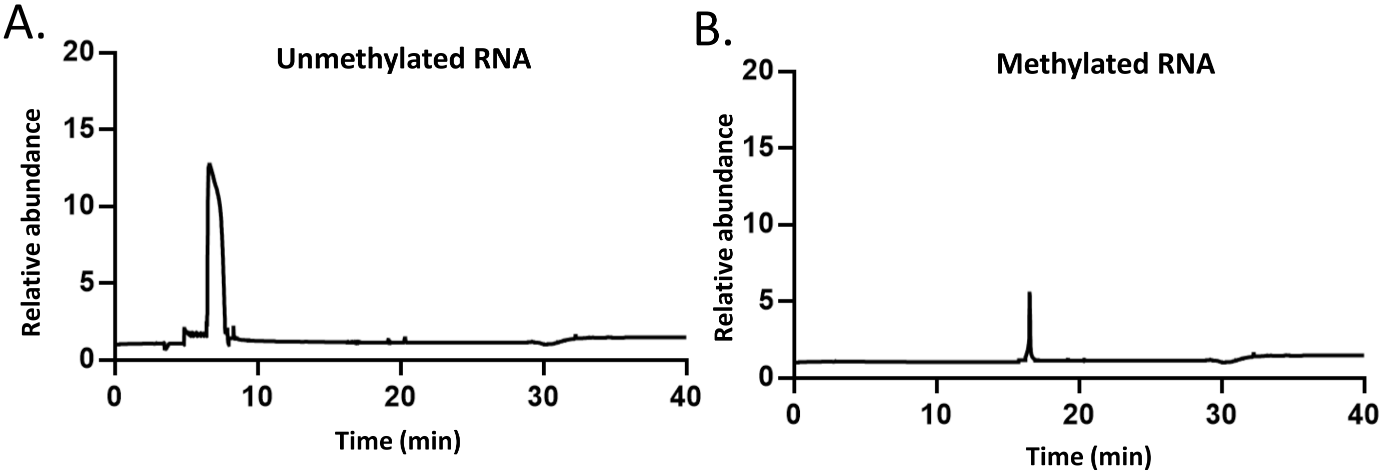

Appendix Figure S1. The HPLC peaks of rRNA. HPLC analysis of unmethylated RNA substrate (A) and methylated RNA standard (B). Both samples were incubated at 37° C for 2 hours, followed by heating at 95° C for 5 minutes prior to analysis.

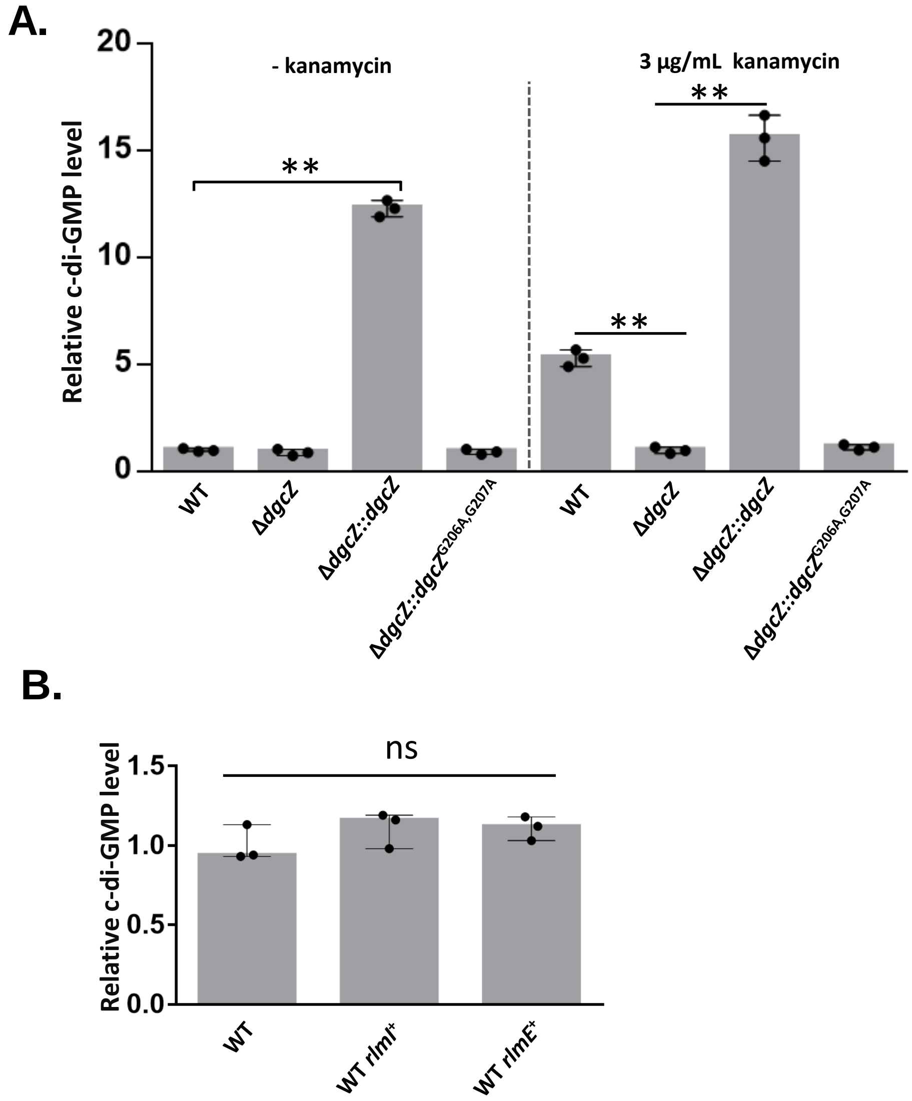

Appendix Figure S2. The relative c-di-GMP level in the strains. (A) Relative c-di-GMP level of four strains *i.e.*, WT, Δ*dgcZ*, Δ*dgcZ*::*dgcZ* and Δ*dgcZ::dgcZ*^{G206A,G207A} with or without kanamycin treatment. The intracellular c-di-GMP concentrations were determined by UPLC-IM-MS. The bar chart shows the relative quantification of c-di-GMP with the data points (three preparations, mean ± range; **$p < 0.01$, two-tailed Student's t-test). (B) Relative c-di-GMP level of three strains *i.e.*, WT, WT *rlmI*$^{+}$ and WT *rlmE*$^{+}$. The intracellular c-di-GMP concentrations were determined by UPLC-IM-MS. The bar chart shows the relative quantification of c-di-GMP with the data points (three preparations, mean ± range; ns: no significant difference, two-tailed Student's t-test).

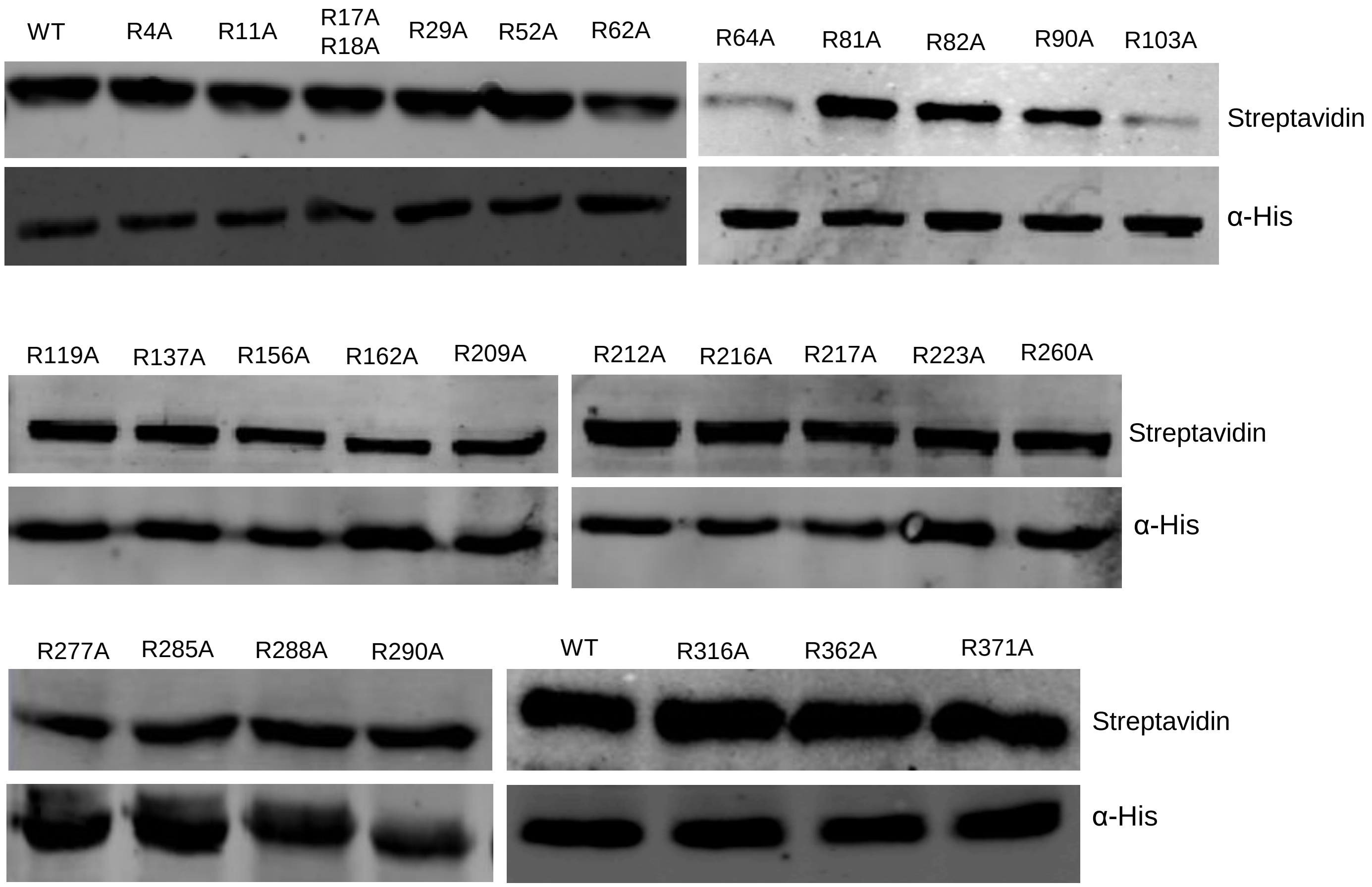

Appendix Figure S3 Streptavidin blotting assays comparing WT and mutant RlmI. The arginine within RlmI were mutated to alanine, and the interaction with c-di-GMP was determined. Streptavidin represents the interaction signals, and α-His represents the protein levels.

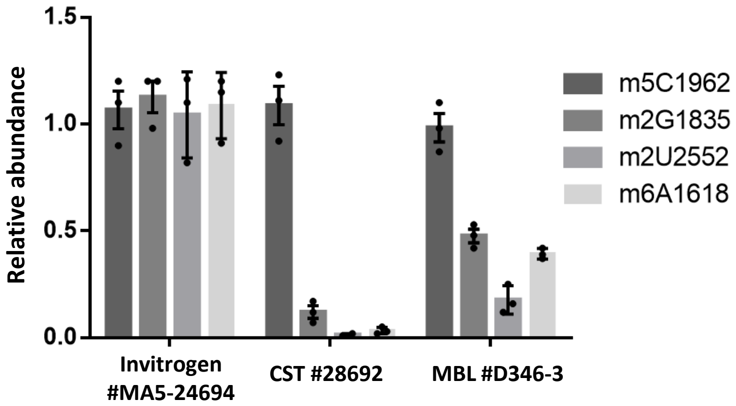

Appendix Figure S4 Comparison of the enrichment specificity of three antibodies against m5C1962. To assess their selectivity, m2G1835, m2U2552, and m6A1618 were included as analogous modifications. The results demonstrate the degree to which each antibody preferentially enriches m5C1962 over the other tested modifications. (n = 3 biological replicates, mean ± s.e.m.)

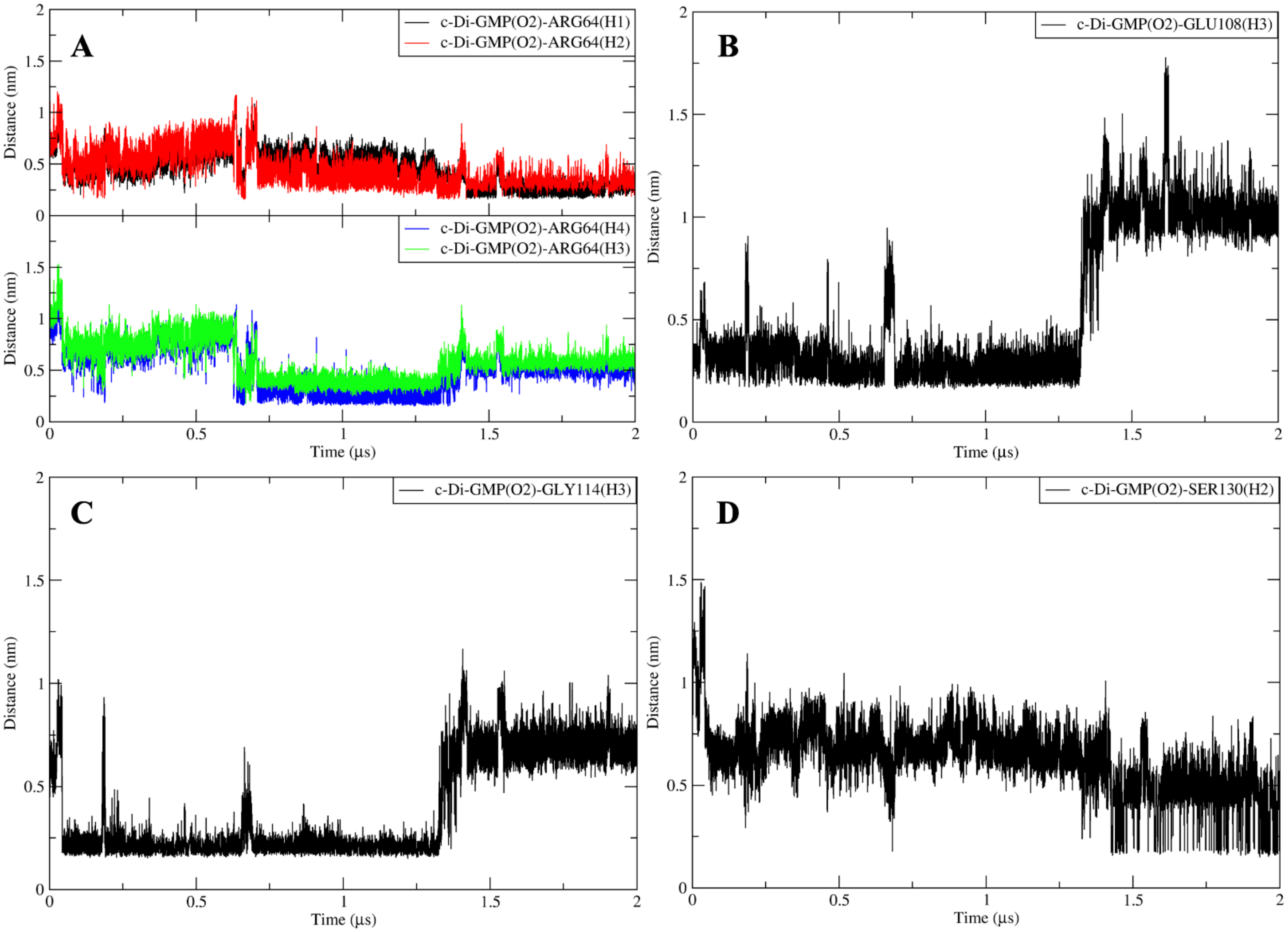

Appendix Figure S5. Atom-atom distances data for hydrogen bond interaction map of c-di-GMP with RlmI. (A) c-di-GMP(O2) interaction with ARG64; (B) c-di-GMP(O2) interaction with GLU108; (C) c-di-GMP(O2) interaction with GLY114; (D) c-di-GMP(O2) interaction with SER130.

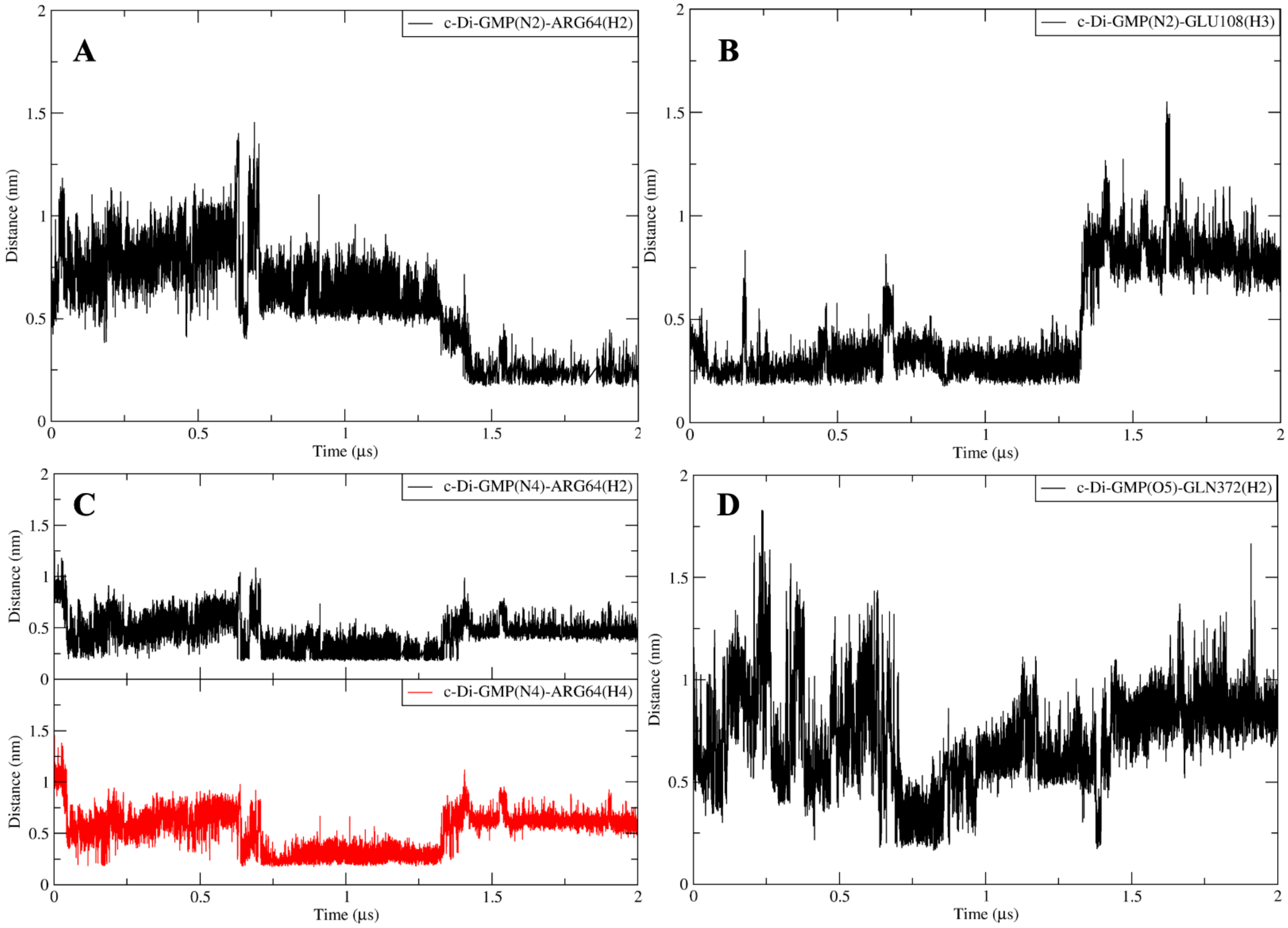

Appendix Figure S6. Atom-atom distances data for hydrogen bond interaction map of c-di-GMP with RlmI. (A) c-di-GMP(N2) interaction with ARG64; (B) c-di-GMP(N2) interaction with GLU108; (C) c-di-GMP(N4) interaction with ARG64; (D) c-di-GMP(O5) interaction with GLN372.

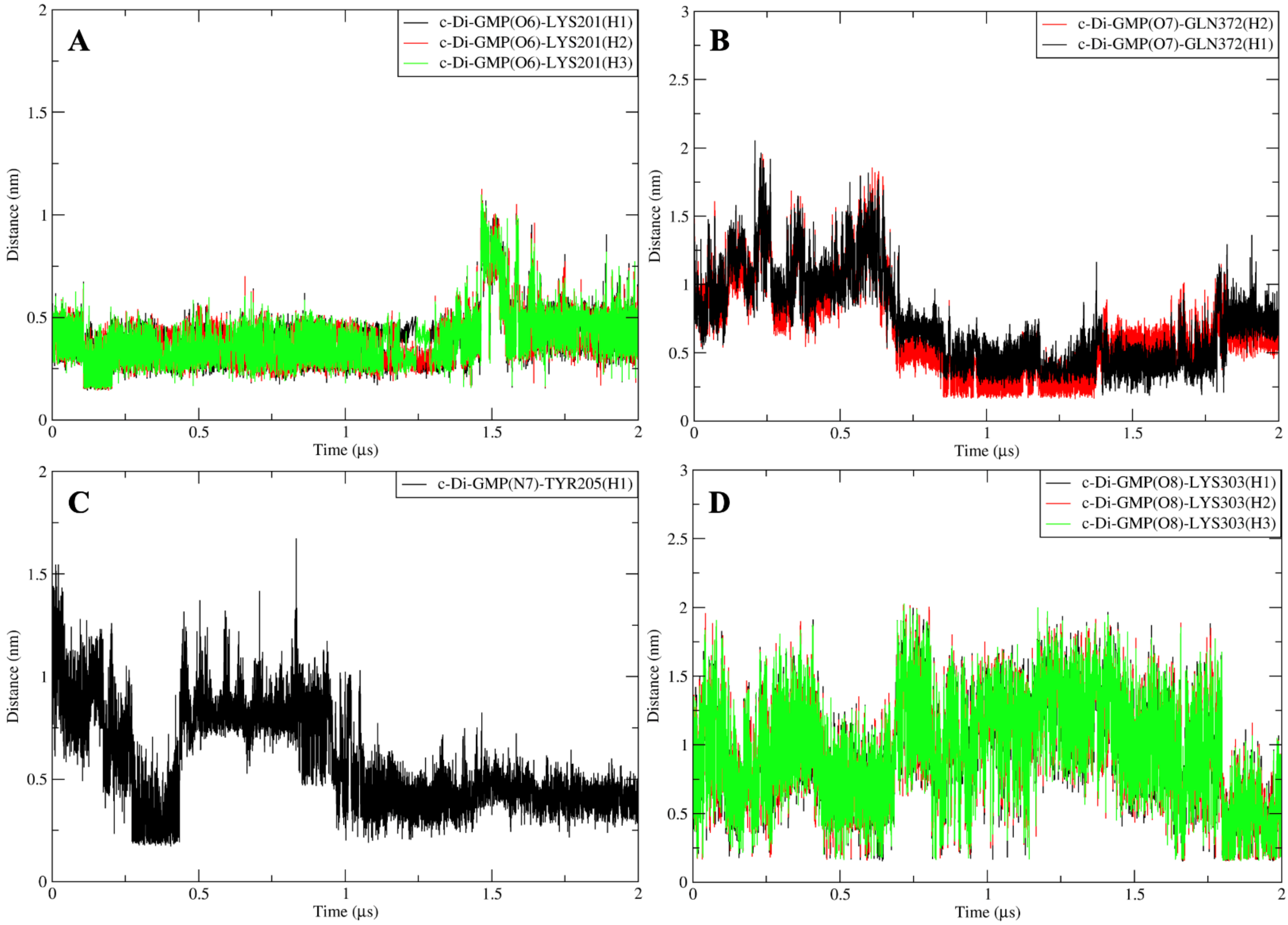

Appendix Figure S7. Atom-atom distances data for hydrogen bond interaction map of c-di-GMP with RlmI. (A) c-di-GMP(O6) interaction with LYS201; (B) c-di-GMP(O7) interaction with GLN372; (C) c-di-GMP(N7) interaction with TYR205; (D) c-di-GMP(O8) interaction with LYS303.

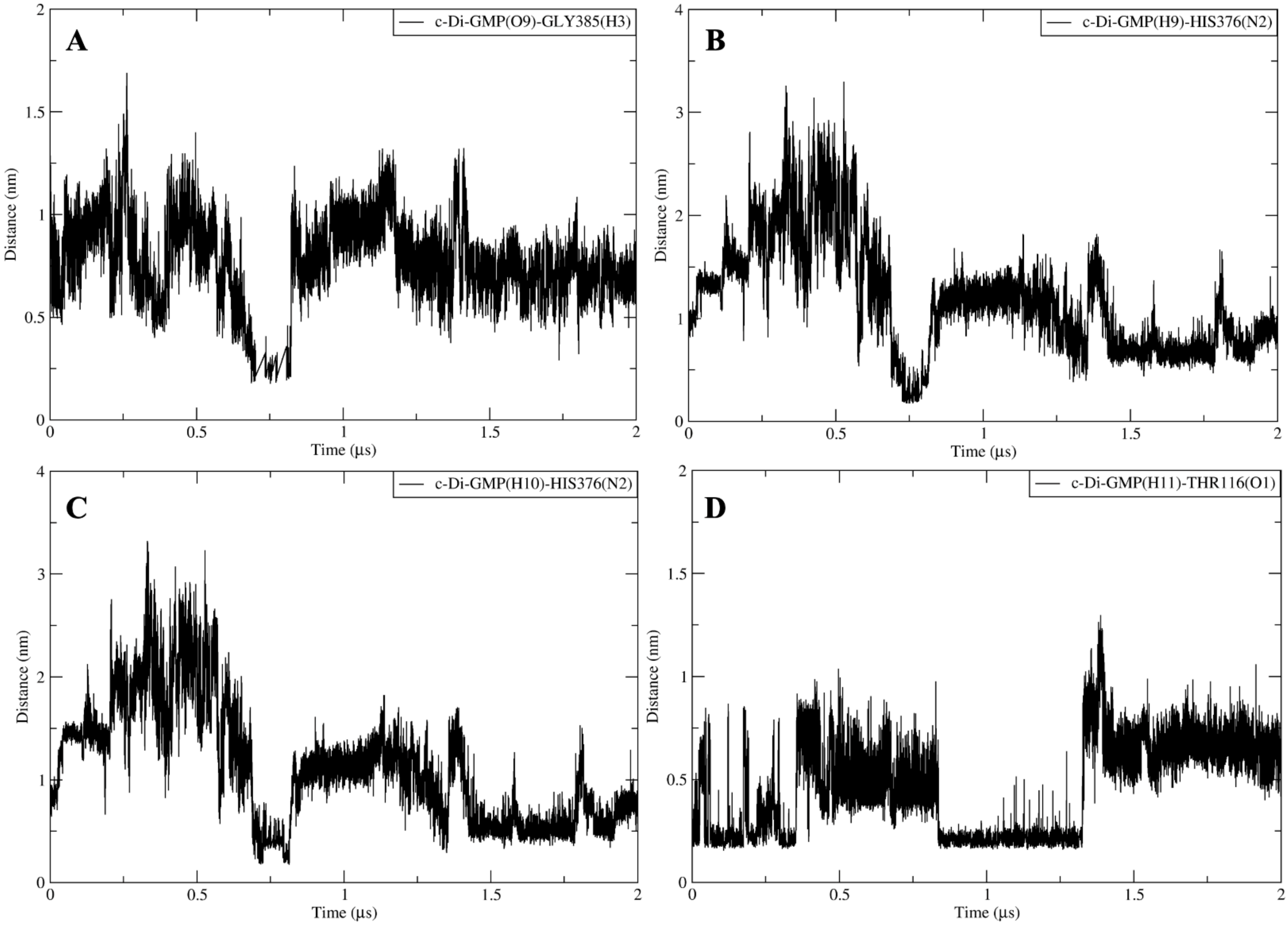

Appendix Figure S8. Atom-atom distances data for hydrogen bond interaction map of c-di-GMP with RlmI. (A) c-di-GMP(O9) interaction with GLY385; (B) c-di-GMP(O9) interaction with HIS376; (C) c-di-GMP(H10) interaction with HIS376; (D) c-di-GMP(H11) interaction with THR116.

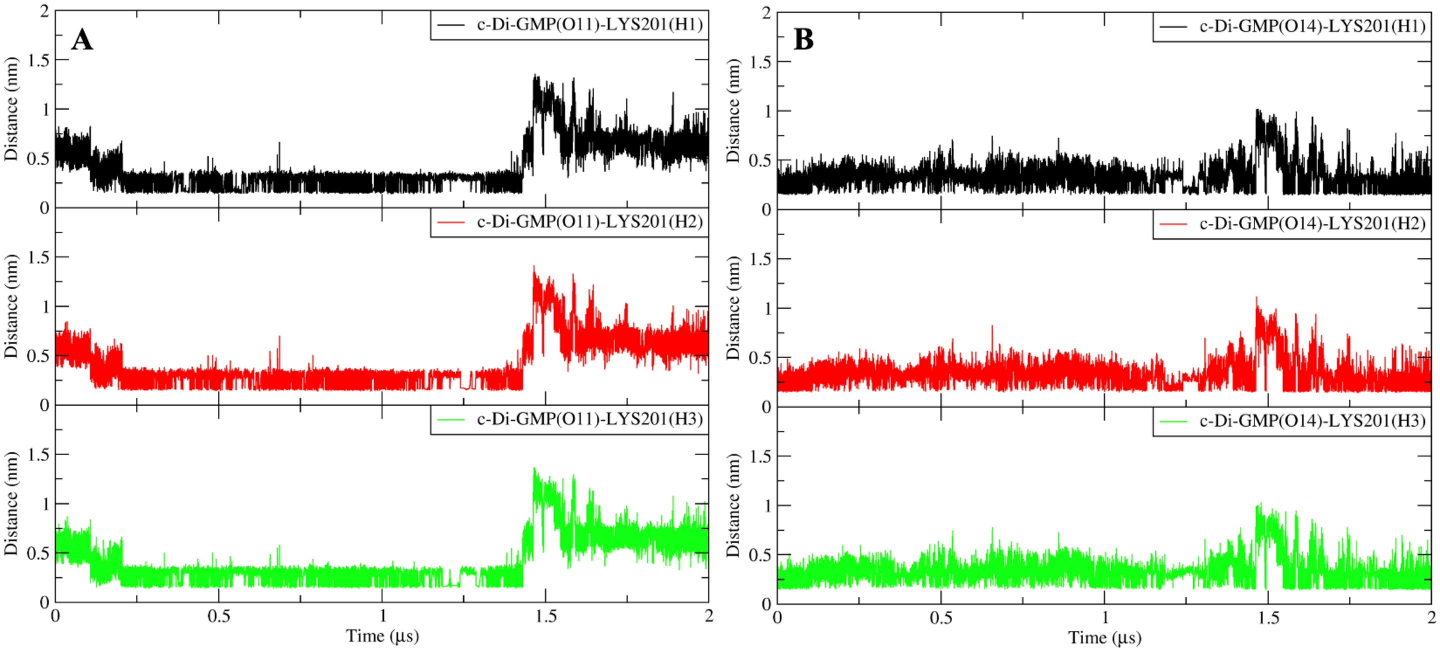

Appendix Figure S9. Atom-atom distances data for hydrogen bond interaction map of c-di-GMP with RlmI. (A) c-di-GMP(O11) interaction with LYS201; (B) c-di-GMP(O14) interaction with LYS201.

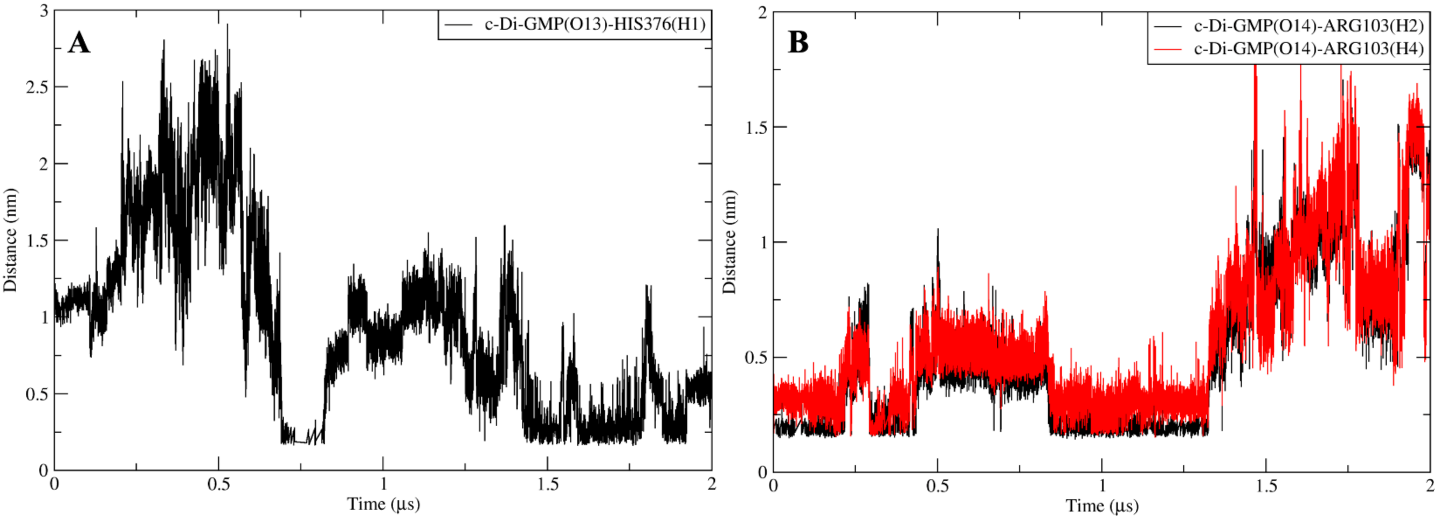

Appendix Figure S10. Atom-atom distances data for hydrogen bond interaction map of c-di-GMP with RlmI. (A) c-di-GMP(O13) interaction with HIS376; (B) c-di-GMP(O14) interaction with ARG103.

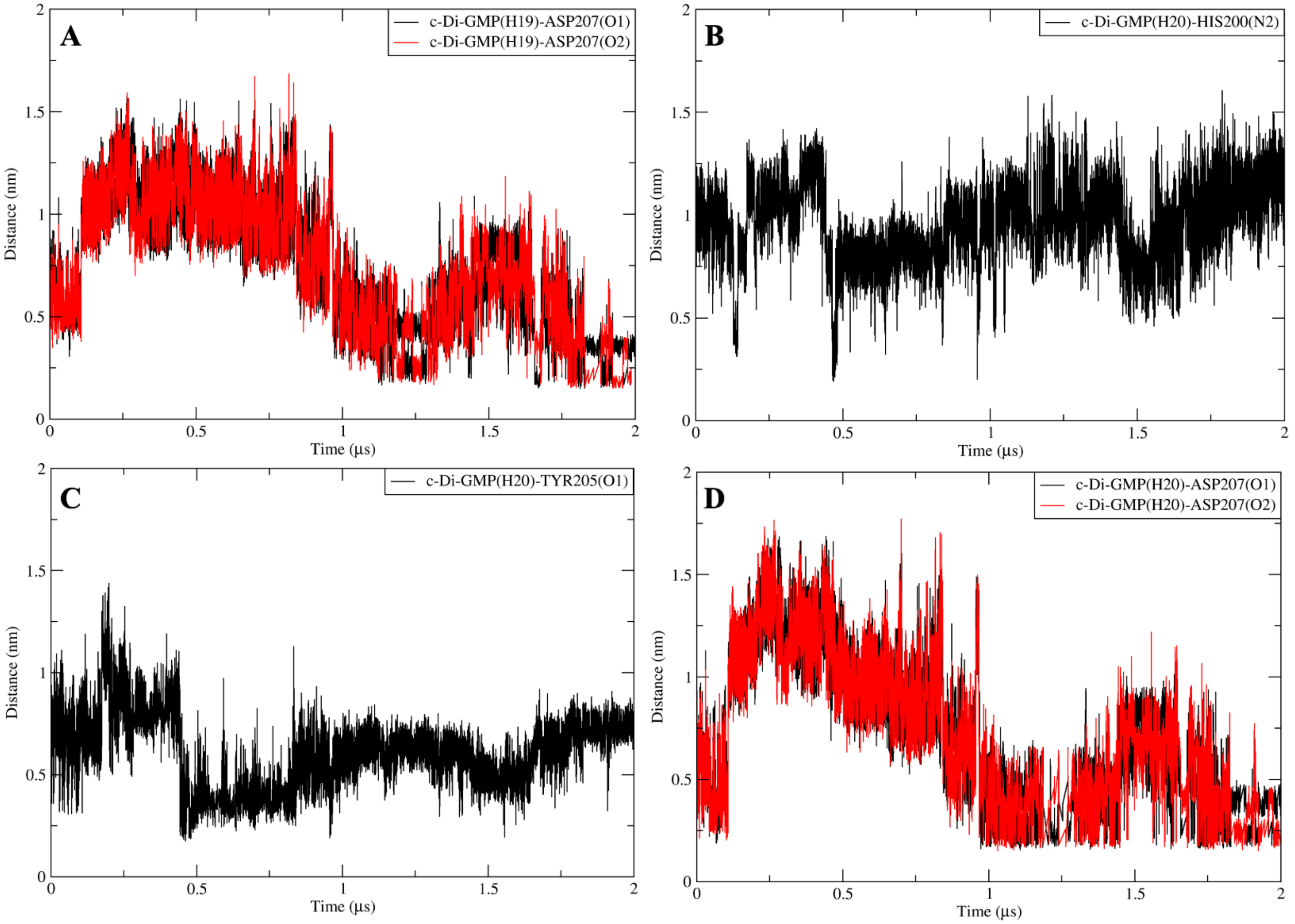

Appendix Figure S11. Atom-atom distances data for hydrogen bond interaction map of c-di-GMP with RlmI. (A) c-di-GMP(H19) interaction with ASP207; (B) c-di-GMP(H20) interaction with HIS200; (C) c-di-GMP(H2) interaction with TYR205; (D) c-di-GMP(H20) interaction with ASP207.

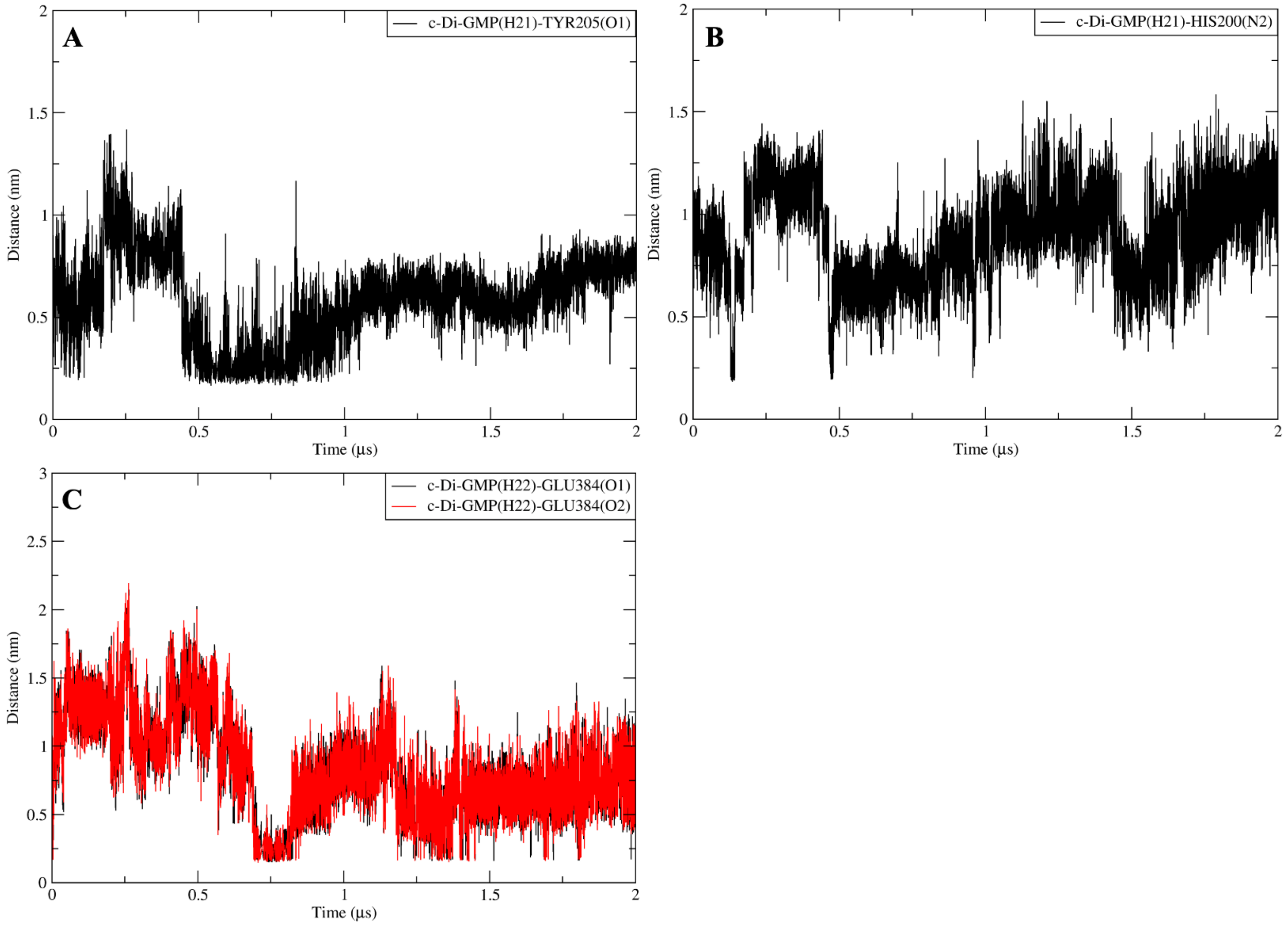

Appendix Figure S12. Atom-atom distances data for hydrogen bond interaction map of c-di-GMP with RlmI. (A) c-di-GMP(H21) interaction with TYR205; (B) c-di-GMP(H21) interaction with HIS200; (C) c-di-GMP(H22) interaction with GLU384.

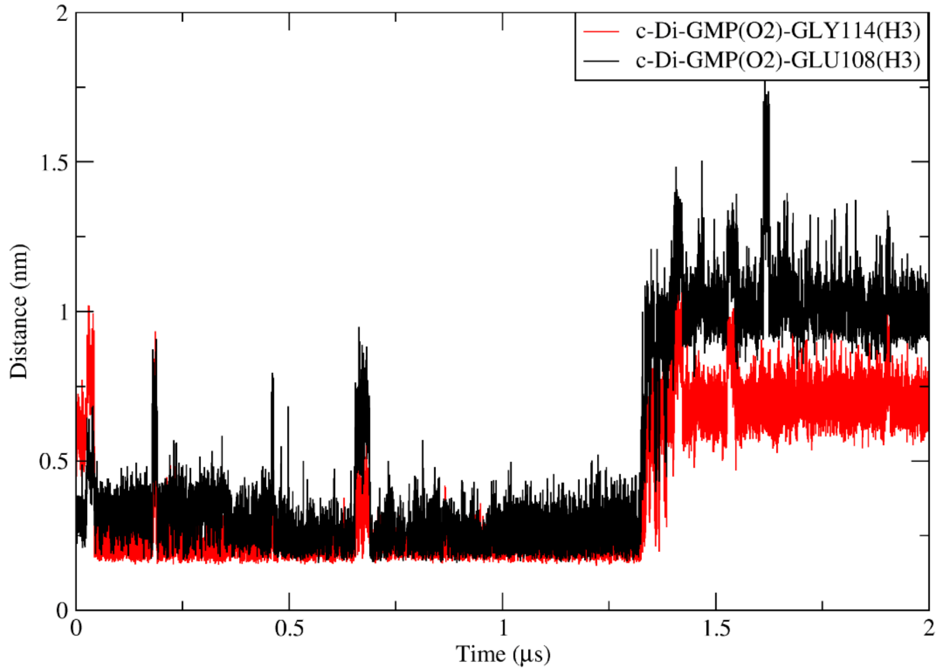

Appendix Figure S13. Comparison of hydrogen bonding interactions of c-di-GMP(O2) with GLY114 and GLU108.

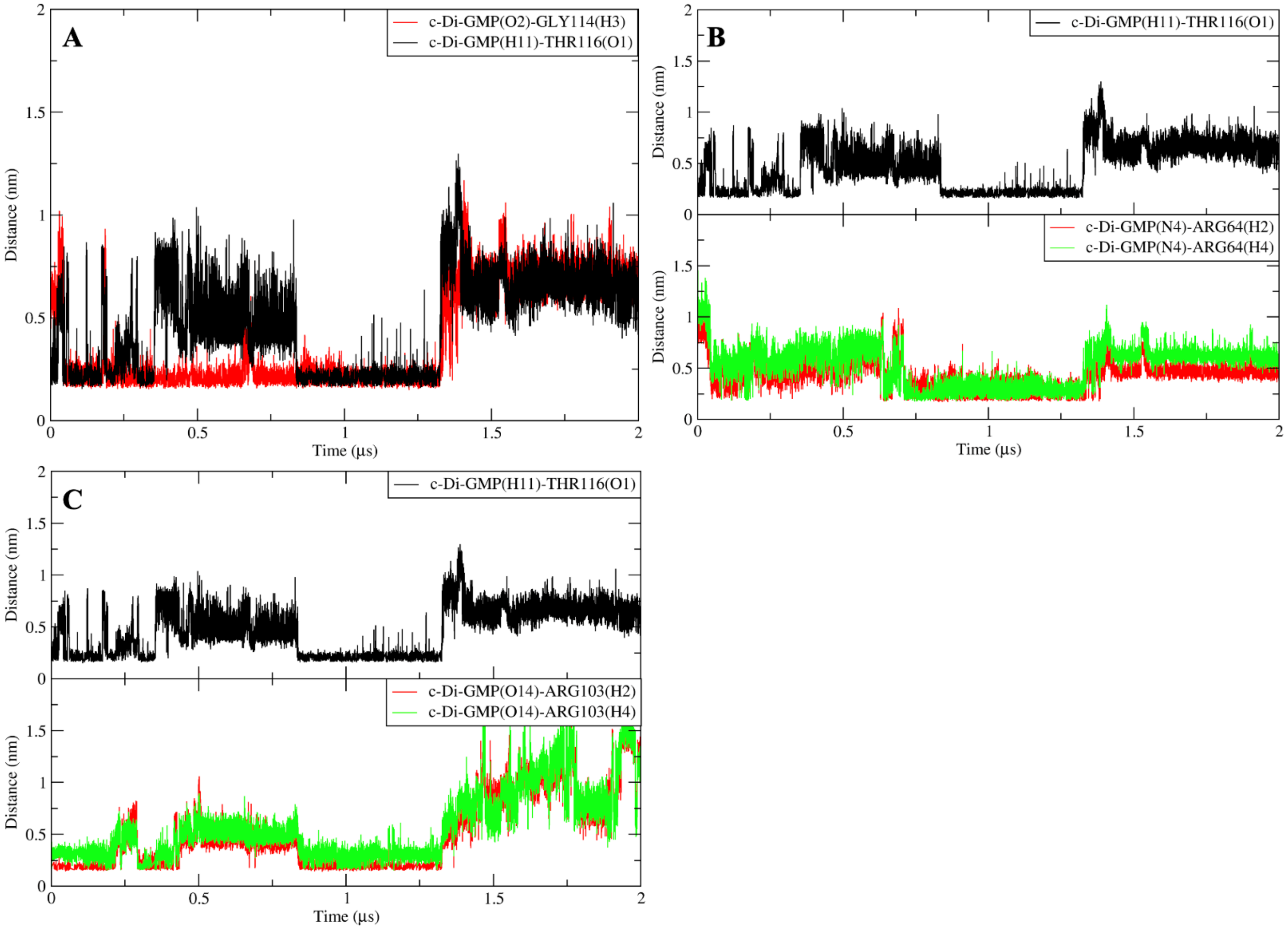

Appendix Figure S14. Comparison of hydrogen bonding interactions of c-di-GMP with THR116, ARG64, ARG103 and GLY114.

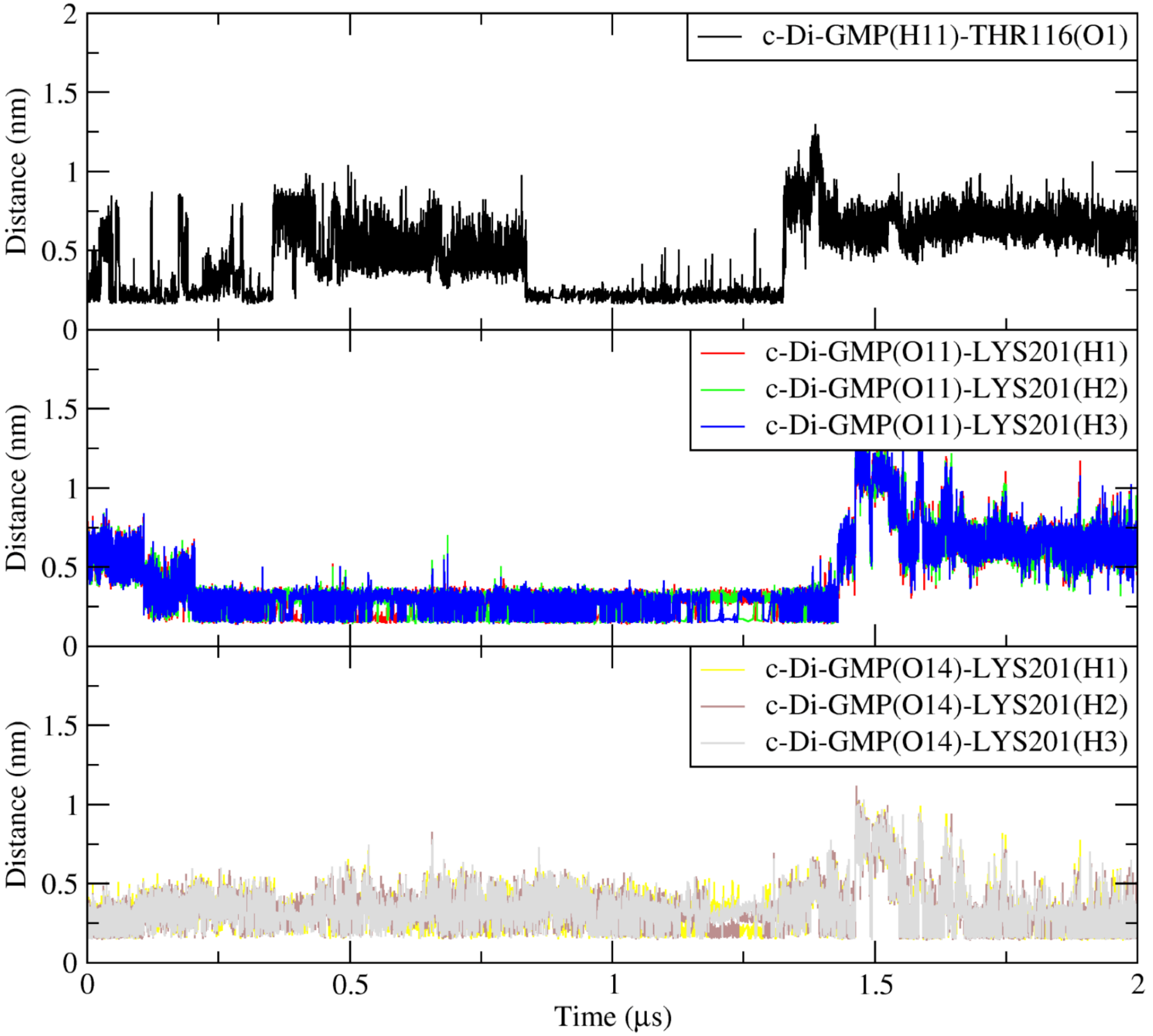

Appendix Figure S15. Comparison of hydrogen bonding interactions of c-di-GMP with THR116 and LYS201.

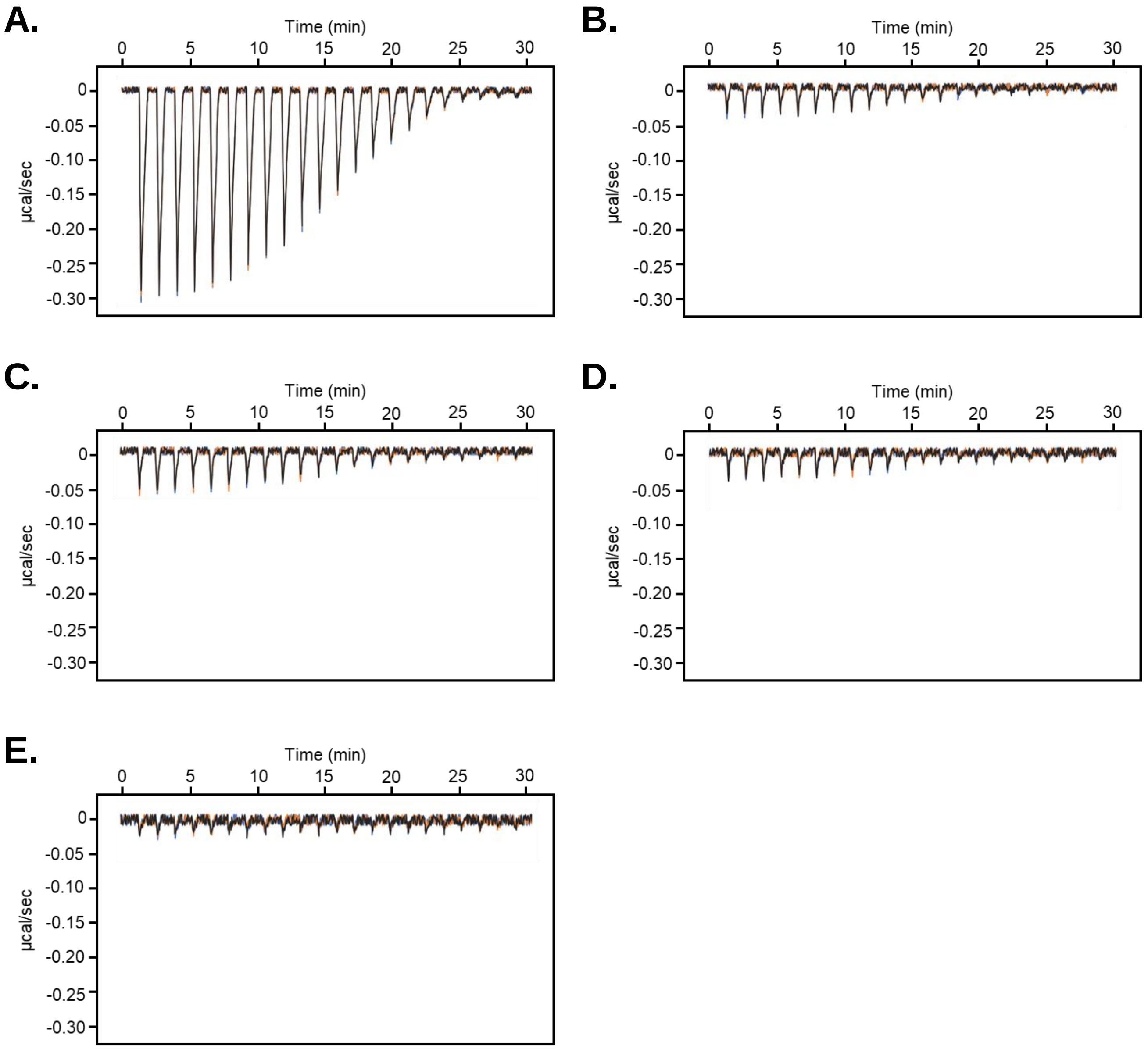

Appendix Figure S16. The Raw data curve for ITC detection of the interaction between RlmI and c-di-GMP. The titration experiment was repeated three times for RlmI (A), $RlmI^{R64A}$ (B), $RlmI^{R103A}$ (C), $RlmI^{G114A}$ (D) and $RlmI^{K201A}$ (E). The curves of the three repeated experiments are marked in black, orange, and blue, respectively.
