# Supplementary figures and images for: c-di-GMP inhibits rRNA methylation and impairs ribosome assembly in the presence of kanamycin
Source: EMBO Rep. 2025 Jan 27;26(5):1367–84. doi: 10.1038/s44319-025-00377-w (PMC11894153; doi:10.1038/s44319-025-00377-w)

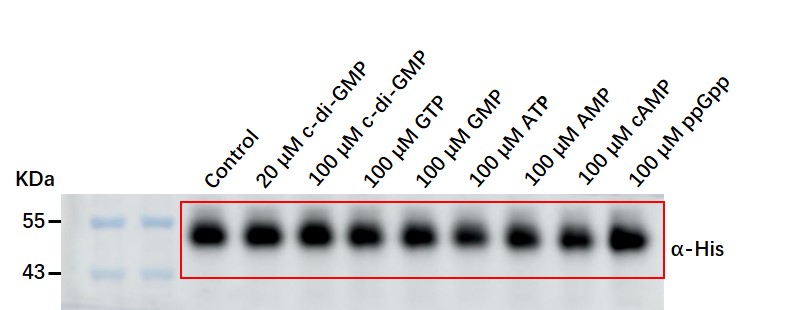

Supplement: Supplementary file 6 — Source data Fig. 2 [file 44319_2025_377_MOESM6_ESM.zip › Source data Figure 2/2C/Fig.2C blot His.jpg]

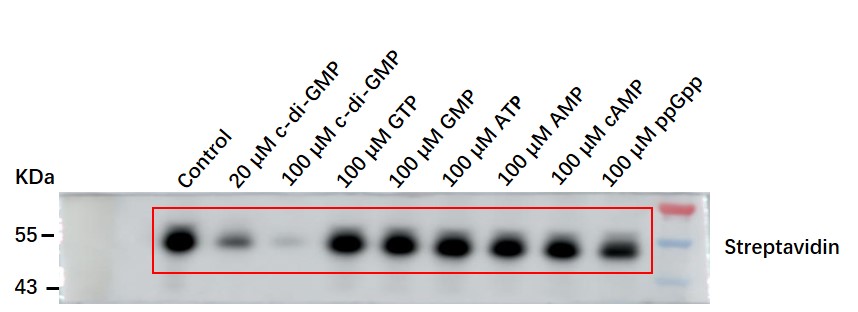

Supplement: Supplementary file 6 — Source data Fig. 2 [file 44319_2025_377_MOESM6_ESM.zip › Source data Figure 2/2C/Fig.2C blot Streptavidin.jpg]

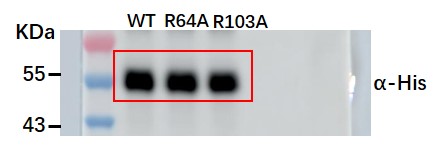

Supplement: Supplementary file 6 — Source data Fig. 2 [file 44319_2025_377_MOESM6_ESM.zip › Source data Figure 2/2D/Fig.2D blot His.jpg]

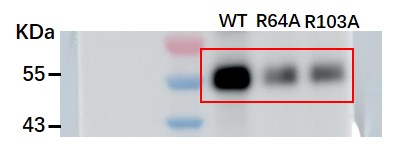

Supplement: Supplementary file 6 — Source data Fig. 2 [file 44319_2025_377_MOESM6_ESM.zip › Source data Figure 2/2D/Fig.2D blot Streptavidin.jpg]

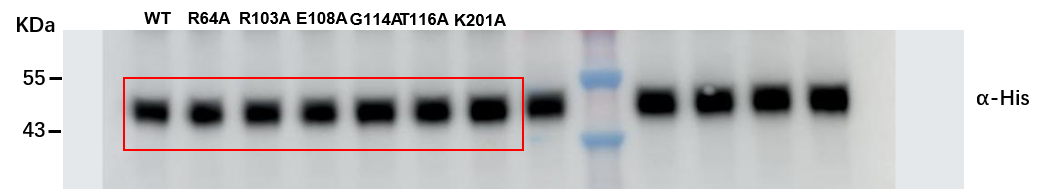

Supplement: Supplementary file 9 — Source data Fig. 5 [file 44319_2025_377_MOESM9_ESM.zip › Source data Figure 5/5D/Fig.4D blot his.png]

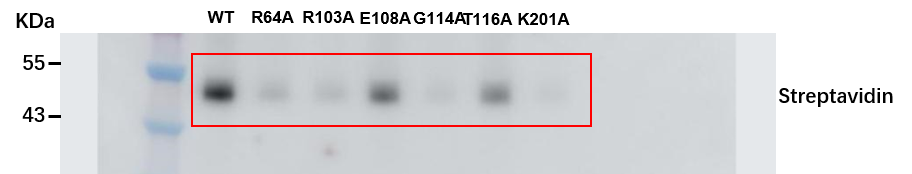

Supplement: Supplementary file 9 — Source data Fig. 5 [file 44319_2025_377_MOESM9_ESM.zip › Source data Figure 5/5D/Fig.4D blot steptavidin.png]

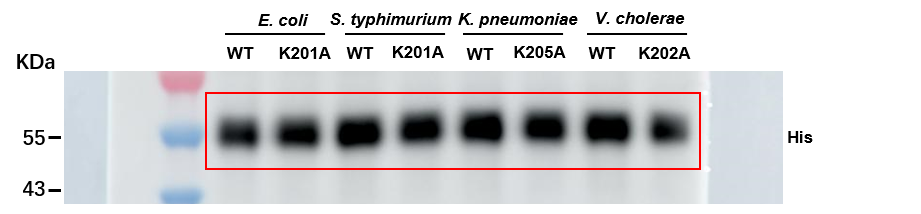

Supplement: Supplementary file 11 — Source data Fig. 7 [file 44319_2025_377_MOESM11_ESM.zip › Source data Figure 7/7B/Fig.6B blot his.png]

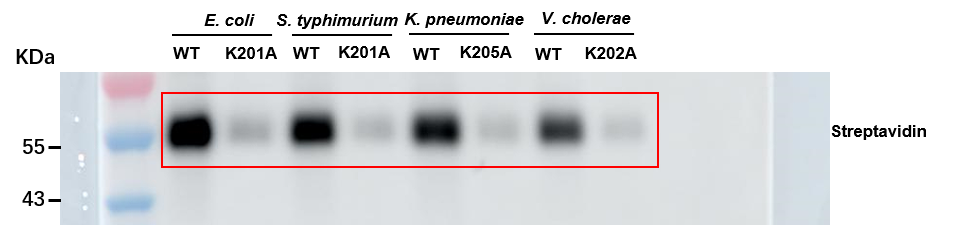

Supplement: Supplementary file 11 — Source data Fig. 7 [file 44319_2025_377_MOESM11_ESM.zip › Source data Figure 7/7B/Fig.6B blot steptavidin.png]
